# A mathematical model for COVID‐19 pandemic—SIIR model: Effects of asymptomatic individuals

**DOI:** 10.1002/jgf2.382

**Published:** 2020-11-01

**Authors:** Masaki Tomochi, Mitsuo Kono

**Affiliations:** ^1^ Department of Economics Okinawa International University Okinawa Japan; ^2^ Faculty of Policy Studies Chuo University Tokyo Japan

**Keywords:** asymptomatic individuals, COVID‐19, finite antibody duration, Health Policy, infectious diseases, SIIR model

## Abstract

A new mathematical model called SIIR model is constructed to describe the spread of infection by taking account of the characteristics of COVID‐19 and is verified by the data from Japan. The following features of COVID‐19: (a) there exist presymptomatic individuals who have infectivity even during the incubation period, (b) there exist asymptomatic individuals who can freely move around and play crucial roles in the spread of infection, and (c) the duration of immunity may be finite, are incorporated into the SIIR model. The SIIR model has the advantage of being able to explicitly handle asymptomatic individuals who are delayed in discovery or are extremely difficult to be discovered in the real world. It is shown that the conditions for herd immunity in the SIIR model become more severe than those in the SIR model; that is, the presence of asymptomatic individuals increases herd immunity threshold (HIT).

## Graphical abstract

A new mathematical model called SIIR is constructed to describe the world‐widely undergoing spread of infection by taking account of the characteristics of COVID‐19 and is verified by the number of confirmed cases in Japan. The SIIR model has the advantage of being able to explicitly handle asymptomatic individuals who are delayed in discovery or are extremely difficult to be discovered in the real world.

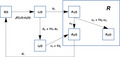

## 1 INTRODUCTION

A history of mankind is a history of fighting against infectious diseases, such as smallpox, polio, plague, flu, AIDS, SARS, and MARS. Because of the spread of COVID‐19 in these days, human beings continue to be suffered by pandemics, that is, danger of collapse of medical, social, and economic system.

Mathematical models have been used to assess the spread of infectious diseases for long time. As infectious diseases change and become more complex, mathematical models have also evolved. Among these models, SIR (susceptible–infectious–recovered [removed]) model, which has three transitioning stages: susceptibility, infectious, and recovered (and removed from the infectious network), has been widely used for discussing the process of infectious diseases spreading. In addition, SEIR model, which divides the infection stage in the SIR model into two stages, noninfectious (exposed) and infectious, and investigates the four transitioning stages, has also been widely used.^1^, ^2^

Since the mechanism of the spread of COVID‐19 consists of direct and indirect contacts between infected and susceptible individuals, it seems that the conventional models sufficiently explain the phenomena if COVID‐19 is regarded to be the same as the conventional infectious diseases. However, unique features of COVID‐19 should be taken into account. These features are (a) there exist presymptomatic individuals who are infectious even during the incubation period, (b) there exist asymptomatic individuals who can move around freely and spread infection, making it difficult to control the situation, and (c) the duration of immunity may be finite and susceptible population is reproduced; that is, it may take long time to achieve herd immunity and several peaks of the spread of infection may occur.

In the next section, SIIR model is introduced to explain the mechanism of COVID‐19 infection spread which cannot be fully explained by the conventional mathematical models such as SIR model and SEIR model. In Section 3, SIIR model is verified by data on Japan to show that the model is suitable. Discussions are given in the last section.

## 2 SIIR MODEL

### 2.1 Model equations

As one of the features of COVID‐19, there is incubation period after infection and presymptomatic carriers can spread the infection during the incubation period. Further, even though the symptomatic carriers or those who are confirmed to be infected are quarantined, there are also asymptomatic individuals who do not develop after the end of the incubation period. This makes the spread of infection difficult to be controlled because asymptomatic individuals are left unchecked without isolation. COVID‐19 is characterized by two different infectious states (presymptomatic and asymptomatic) and is pointed out that antibody duration is not so long.^10^ These are not considered in SIR or SEIR models and, therefore, a model that takes such characteristics of COVID‐19 into account is needed.

**Figure 1:**
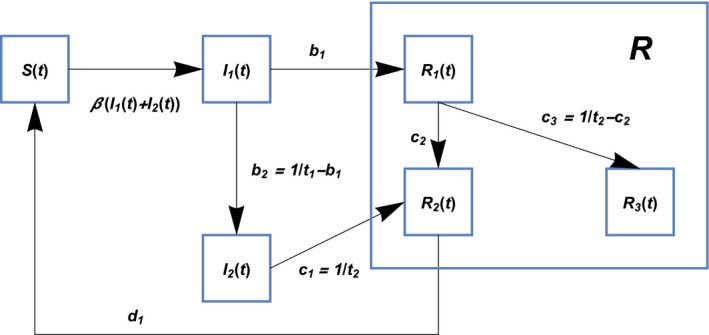
Structure of the SIIR model: *S*(*t*) as susceptible population, *I*_1_(*t*) as presymptomatic population (infectious), *I*_2_(*t*) as asymptomatic population (infectious), *R*_1_(*t*) as symptomatic population (quarantined and not infectious), *R*_2_(*t*) as recovered population (recovered with antibody and not infectious), and *R*_3_(*t*) as fatalities because of COVID‐19 (not infectious)

Here, parties concerned in SIIR model at time *t* are *S*(*t*) as susceptible population, *I*_1_(*t*) as presymptomatic population (infectious), *I*_2_(*t*) as asymptomatic population (infectious), *R*_1_(*t*) as symptomatic population (not infectious by quarantine), *R*_2_(*t*) as recovered population (with antibody and not infectious), and *R*_3_(*t*) as fatalities because of COVID‐19 (not infectious). Then, the interrelationship among the above variables is shown in Figure 1 and is described by the following coupled differential equations:

(1) $$\frac{dS(t)}{\mathrm{dt}}=‐\beta S\left(t\right)\left(I_{1}\left(t\right)+I_{2}\left(t\right)\right)+d_{1}R_{2}\left(t\right),$$

(2) $$\frac{dI_{1}\left(t\right)}{\mathrm{dt}}=\beta S\left(t\right)\left(I_{1}\left(t\right)+I_{2}\left(t\right)\right)‐\left(b_{1}+b_{2}\right)I_{1}\left(t\right),$$

(3) $$\frac{dI_{2}\left(t\right)}{\mathrm{dt}}=b_{2}I_{1}\left(t\right)‐c_{1}I_{2}\left(t\right),$$

(4) $$\frac{dR_{1}\left(t\right)}{\mathrm{dt}}=b_{1}I_{1}\left(t\right)‐\left(c_{2}+c_{3}\right)R_{1}\left(t\right),$$

(5) $$\frac{dR_{2}\left(t\right)}{\mathrm{dt}}=c_{1}I_{2}\left(t\right)+c_{2}R_{1}\left(t\right)‐d_{1}R_{2}\left(t\right),$$

(6) $$\frac{dR_{3}\left(t\right)}{\mathrm{dt}}=c_{3}R_{1}\left(t\right),$$

which is called SIIR model since there are two states in the infection stage, that is, *I*_1_ and *I*_2_.

In the SIIR model, presymptomatic and asymptomatic individuals are at different stages but they are both not quarantined and are free to move around. Therefore, in order to clearly show that they are infectious, they are denoted as *I*_1_ and *I*_2_, respectively. On the other hand, asymptomatic individuals, recovered individuals with antibody, and those who passed away are not infectious, so they are represented by *R*, that is, *R*_1_, *R*_2_, and *R*_3_, respectively. The SIIR model is used to distinguish it from the SIR model. The feature of the model is that infectious and noninfectious states have internal structures.

The coefficient *β* in the SIIR model corresponds to the probability, with which susceptible individuals (*S*) will be infected by contacting with those who are infectious (*I*_1_ + *I*_2_) at a different stage of infectivity, and has a similar role to the one in the SIR model. Presymptomatic individuals (*I*_1_) bifurcate into symptomatic individuals (*R*_1_) who are quarantined and asymptomatic individuals (*I*_2_) who are not quarantined with the coefficients *b*_1_ and *b*_2_, respectively. The meanings of these coefficients are understood in the following way. Assuming presymptomatic population are evenly distributed during the incubation period *t*_1_, out of presymptomatic population *I*_1_(*t*), those at the end of incubation period *t*_1_ denoted by n(*t*_1_) may be expressed by n(*t*_1_) = *I*_1_(*t*)/*t*_1_. At the end of the incubation period, the *i*‐th presymptomatic individual bifurcates into the symptomatic state with a probability *b*_1_ *_i_* and asymptomatic state with a probability *b*_2_ *_i_* where *b*_1_ *_i_* + *b*_2_ *_i_* = 1. On the other hand, assuming that symptomatic and asymptomatic individuals are equally distributed in the period of the onset, they are given by *R*_1_(*t*)/*t*_2_ and *I*_2_(*t*)/*t*_2_, respectively, at the time of transition from incubation to onset. Thus, we have,

$$\sum_{i=1}^{n(t_{1})}b_{1i}=\frac{R_{1}\left(t\right)}{t_{2}}=\Delta R_{1}\left(t\right),\;\sum_{i=1}^{n(t_{1})}b_{2i}=\frac{I_{2}\left(t\right)}{t_{2}}=\Delta I_{2}\left(t\right),$$

$$\sum_{i=1}^{n(t_{1})}\left(b_{1i}+b_{2i}\right)=n\left(t_{1}\right)=\frac{I_{1}\left(t\right)}{t_{1}}=\frac{1}{t_{2}}\left(R_{1}\left(t\right)+I_{2}\left(t\right)\right)$$

Since *I*_1_(*t*) bifurcates into ∆*R*_1_(*t*) with *b*_1_ and ∆*I*_2_(*t*) with *b*_2_, we have,

(7) $$\Delta R_{1}\left(t\right)=b_{1}I_{1}\left(t\right),\;\Delta I_{2}\left(t\right)=b_{2}I_{1}\left(t\right),\;\to \;b_{1}+b_{2}=\frac{1}{t_{1}}.$$

Factors that determine whether those who are infected become symptomatic or asymptomatic have not yet been identified. However, if health factors are not considered, 1/*t*_1_ is apportioned between *b*_1_ and *b*_2_.

The coefficients *c*_1_, *c*_2_, and *c*_3_ are also related to the onset period. At the end of the onset period *t*_2_, the following,

$$\frac{1}{t_{2}}R_{1}\left(t\right)=\Delta_{1}R_{2}\left(t\right)+\Delta R_{3}\left(t\right),$$

holds at the transition from symptomatic to recovery with antibody or to death. For the transition from asymptomatic to recovery with antibody, the following relation holds:

$$\frac{1}{t_{2}}I_{2}\left(t\right)=\Delta_{2}R_{2}\left(t\right),$$

as long as there is no transition from asymptomatic to death. Therefore, we have,

$$\Delta_{1}R_{2}\left(t\right)=c_{2}R_{1}\left(t\right),\;\Delta_{2}R_{2}\left(t\right)=c_{1}I_{2}\left(t\right),\;\Delta R_{3}=c_{3}R_{1}\left(t\right),$$

which leads to,

$$c_{1}=c_{2}+c_{3}=\frac{1}{t_{2}}.$$

That is, *c*_1_ is the inverse of the onset period, and 1/*t*_2_ is apportioned between *c*_2_ and *c*_3_.

If recovered individuals *R*_2_ lose their antibody at time *t*, they become susceptible *S* again, and the coefficient *d*_1_ represents the inverse of the antibody duration.

In this way, the SIIR model, in spite of a continuous model, is explicitly to capture the duration of each stage. In addition, the SIIR model is a five‐variable system because of the following conserved quantity given by,

$$\frac{d}{\mathrm{dt}}\left(S\left(t\right)+I_{1}\left(t\right)+I_{2}\left(t\right)+R_{1}\left(t\right)+R_{2}\left(t\right)+R_{3}\left(t\right)\right)=0\;\to \;N=S\left(t\right)+I_{1}\left(t\right)+I_{2}\left(t\right)+R_{1}\left(t\right)+R_{2}\left(t\right)+R_{3}\left(t\right).$$

If the variables are normalized by the conserved quantity *N*, it is convenient to see the characteristic behaviors common to the system with different *N*. In verifying the model with data, the parameters and scale conversion with respect to *N* are required when the theoretical results best‐fit the data. For example, for the spread of infection in Japan, it does not make sense to set the question of how the entire population of Japan of one hundred twenty‐six million susceptible individuals is infected. This is because the infection cannot evenly spread throughout Japan. There are many high‐risk areas such as densely populated areas and workplaces scattered throughout Japan. Since the spread of infection in each place is similar to each other, by combining the results of the normalized model as a whole of local infections such as cluster infections, community‐acquired infections, and domestic infections, the substance of the spread of global infection may become clear. This is the power of mathematical models. So, we normalize the variables in the SIIR model by *N* and make the following replacements:

$$\left\{\frac{S(t)}{N},\frac{I_{i}\left(t\right)}{N},\frac{R_{i}\left(t\right)}{N}\right\}\;\to \;\left\{S\left(t\right),I_{i}\left(t\right),R_{i}\left(t\right)\right\},\;\beta N\;\to \;\beta .$$

By these replacements, Equations from (1) to (6) representing the SIIR model are isomorphic even after normalization.

### 2.2 Numerical solutions of SIIR model

**Figure 2:**
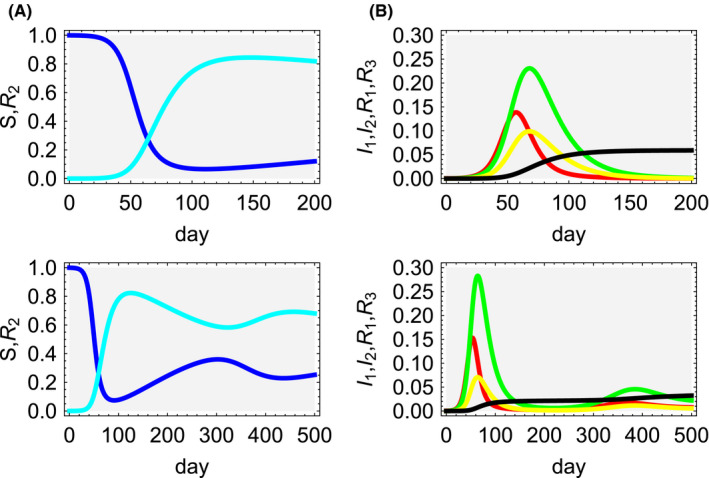
Single peak (upper row) and multiple peak (lower row) solution. Upper and lower left: the susceptible population *S*(*t*) (blue) and recovered population with antibody *R*_2_(*t*) (cyan). Upper and lower right: The presymptomatic population *I*_1_(*t*) (red), asymptomatic population *I*_2_(*t*) (green), symptomatic and quarantined population *R*_1_(*t*) (yellow), and fatalities *R*_3_(*t*) (black)

Now, we look for numerical solutions of the SIIR model. Figure 2 (single peak for the upper row and multiple peaks for the lower row) shows the time evolution of the variables of the SIIR model where the parameters and initial values are set as follows:

$$N=1,\;t_{1}=5,\;t_{2}=17,\;\beta =0.2,$$

$$b_{1}=\frac{0.3}{t_{1}},\;b_{2}=\frac{1}{t_{1}}‐b_{1},\;c_{1}=\frac{1}{t_{2}},\;c_{2}=\frac{0.8}{t_{2}},\;c_{3}=\frac{1}{t_{2}}‐c_{1},$$

$d_{1}=0.001$ (for Figure 2 upper row) and $d_{1}=0.03$ (for Figure 2 lower row) and

$$S\left(0\right)=N‐I_{1}\left(0\right)=S_{0},\;I_{1}\left(0\right)=0.0004,\;I_{2}\left(0\right)=R_{1}\left(0\right)=R_{2}\left(0\right)=R_{3}\left(0\right)=0.$$

As is expected, the susceptible population *S*(*t*) (blue) decreases and the presymptomatic population *I*_1_(*t*) (red) increases, which is followed by the growth of both the asymptomatic population *I*_2_(*t*) (green) and the symptomatic and quarantined population *R*_1_(*t*) (yellow). Then, the recovered population with antibody *R*_2_(*t*) (cyan) and the fatalities *R*_3_(*t*) (black) increase to certain saturation levels. Since we set *b*_2_ > *b*_1_, the asymptomatic population (green) becomes larger than the symptomatic and quarantined population (yellow). In this way, the SIIR model has a definite advantage of handling asymptomatic population which is extremely difficult to be discovered in societies and plays crucial roles in infection spreading. Moreover, because *d*_1_ ≠ 0, for *t* ≫ 1/*d*_1_, the recovered population with antibody (cyan) gradually decreases and the susceptible population (blue) gradually increases. For large *t,* the susceptible population remains finite, showing realization of herd immunity which is discussed in Section 2.4.

During the intermediate transition state of the entire process of infection spreading and termination, the presymptomatic population, symptomatic and quarantined population, and asymptomatic population have their own peaks in the short term. It is *β* and *t*_1_ that constitute the basic reproduction number and determine rising speed of the peak of the presymptomatic population. If *β* is fixed, the larger *t*_1_, the faster presymptomatic population rises, the peak position shifts to the smaller time axis, and the larger the value of the peak. This is because if *t*_1_ is larger, presymptomatic individuals stay in incubation state for a longer time, and conversely, if *t*_1_ is smaller, they immediately move to the next stage. Therefore, the peak width of the presymptomatic population depends on the magnitude of *t*_1_. The presymptomatic individuals bifurcate to the symptomatic individuals with the ratio of *b*_1_ and asymptomatic individuals with *b*_2_ = 1/*t*_1_ − *b*_1_. Therefore, when *b*_1_ is smaller, asymptomatic population increases, leading to a larger feedback to presymptomatic population which in turn grows faster with higher peak and narrower width. On the other hand, when *b*_1_ becomes larger, the symptomatic and quarantined population increases, the feedback to presymptomatic population becomes smaller, resulting in slower growth, smaller peak, and wider width. Thus, during the intermediate transition states, *b*_1_ determines position, growth rate, and width of the peak of infectious population.

The *t*_2_ determines how symptomatic and asymptomatic population decline from the peak. The reason is clear from the terms *c*_2_ = 1/*t*_2_ and *c*_2_ + *c*_3_ = 1/*t*_2_ of Equations (3) and (4). If *t*_2_ becomes larger, the attenuation becomes gentler and the peak width becomes wider.

In the SIIR model, all recovered individuals have antibodies, but since the antibody duration is set as 1/*d*_1_, they become susceptible again over time, that is, small peaks will be repeated until complete termination of the infection.

### 2.3 Basic reproduction number and effective reproduction number for SIIR model

Basic reproduction number of the SIIR model is very much different from that of the SIR model. In the SIR model, $R_{0}$ > 1 is a condition for infection to be spread. However, in Figure 2 for the SIIR model, even though we have,

$$R_{0}=\frac{\beta S_{0}}{b_{1}+b_{2}}=\beta S_{0}t_{1}=0.9996(<1),$$

spread of infection has still occurred. This is because the condition on $R_{0}$ in the SIIR model is relaxed because of the presence of asymptomatic individuals, which is one of the characteristics of the SIIR model. This is understood from the initial behavior of the SIIR model. Since *S*(*t*) ≃ *S*_0_ can be used near *t* ≃ 0, Equations (2) and (3) are expressed as,

$$\frac{d}{\mathrm{dt}}\left(\begin{array}{c} I_{1}(t)\\ I_{2}(t) \end{array}\right)=A\left(\begin{array}{c} I_{1}(t)\\ I_{2}(t) \end{array}\right),\;A=\left(\begin{array}{cc} \beta S_{0}‐(b_{1}+b_{2}) & \beta S_{0}\\ b_{2} & ‐c_{1} \end{array}\right).$$

The eigenvalue *λ* of *A* is calculated as,

$$\lambda =\frac{1}{2}\{‐\{(b_{1}+b_{2})(1‐R_{0})+c_{1}\}\;\pm \sqrt{\left\{(b_{1}+b_{2})(1‐R_{0})+c_{1}\}{\;}^{2}‐4(b_{1}+b_{2})\{c_{1}‐(c_{1}+b_{2})R_{0}\right\}}\},$$

from which the condition *λ* > 0 gives,

(8) $$R_{0}>\frac{c_{1}}{c_{1}+b_{2}}.$$

In Equation (8), the condition for the spread of infection in SIR model is recovered for *b*_2_ = 0. The right‐hand side of Equation (8) is smaller than 1 for *b*_2_ > 0. Thus, the presence of asymptomatic individuals reduces the threshold of $R_{0}$ and the spread of infection is taken place even for $R_{0}$ < 1.

The effective reproduction number $R_{e}\left(t\right)$ for the SIIR model is calculated from Equation (2) as follows:

$$\frac{dI_{1}\left(t\right)}{\mathrm{dt}}=\left(b_{1}+b_{2}\right)\left(R_{e}\left(t\right)‐1\right)I_{1}\left(t\right),\;R_{e}\left(t\right)=\frac{\beta }{b_{1}+b_{2}}S\left(t\right)\frac{I_{1}\left(t\right)+I_{2}\left(t\right)}{I_{1}\left(t\right)}.$$

Since the basic reproduction number $R_{0}$ for the SIIR model is specific to the infectious disease and is determined based on the initial value as,

(9) $$R_{0}=\frac{\beta }{b_{1}+b_{2}}S_{0},\;S_{0}=S\left(0\right),$$

the effective reproduction number is represented as,

(10) $$R_{e}\left(t\right)=R_{0}\frac{S(t)}{S_{0}}\left(1+\frac{I_{2}\left(t\right)}{I_{1}\left(t\right)}\right).$$

The SIIR model cannot be solved analytically but can be solved numerically, and numerical solutions are used to evaluate Equation (10).

Equation (8) is also obtained based on Equation (10). Using a resolvent operator:

$$L\left(c_{1}\right)=\frac{d}{\mathrm{dt}}+c_{1},$$

Equation (3) has the following formal solution:

$$I_{2}\left(t\right)=b_{2}L^{‐1}\left(c_{1}\right)I_{1}\left(t\right).$$

Near t=0, we have,

$$L^{‐1}\left(c_{1}\right)∼\frac{1}{c_{1}},$$

leading to

$$R_{e}=R_{0}\left(1+\frac{b_{2}}{c_{1}}\right).$$

The condition $R_{e}$ > 1 gives Equation (8). Thus, the effective reproduction number depends on both the incubation period *t*_1_ = 1/(*b*_1_ + *b*_2_) and the onset period *t*_2_ = 1/*c*_1_.

### 2.4 Condition for herd immunity

In order for the spread of infection to reach an end, we have $R_{e}\left(t\right)$ < 1, which is equivalent to the condition for the onset of herd immunity:

(11) $$1‐\frac{S(t)}{S_{0}}>1‐\frac{1}{R_{0}}\frac{I_{1}\left(t\right)}{I_{1}\left(t\right)+I_{2}\left(t\right)}>1‐\frac{1}{R_{0}}.$$

If *S*(*t*) is a monotonically decreasing function which is the case for *d*_1_ = 0, the condition for the herd immunity of the SIIR model is shown to be more severe than that of the SIR model, and the presence of asymptomatic individuals increases herd immunity threshold (HIT). In addition, since those who lose antibody after recovery are transferred to be susceptible again, *S*(*t*) may not be a monotonically decreasing function, for which $R_{e}\left(t\right)$ oscillates around 1 and the infection peaks will appear multiple times in a period of 1/*d*_1_, the antibody duration.

### 2.5 Cumulative fatalities of SIIR model

It is very much important to examine fatalities caused by infectious diseases. Here, we evaluate cumulative fatalities in the SIIR model. As we see earlier, we have,

$$c_{1}=c_{2}+c_{3},$$

and, from Equations (3) and (4), we have,

$$\left(\frac{d}{\mathrm{dt}}+c_{1}\right)\left(I_{2}(t)‐\frac{b_{2}}{b_{1}}R_{1}(t)\right)=0,\;\to \;I_{2}(t)‐\frac{b_{2}}{b_{1}}R_{1}(t)=\left\{I_{2}(0)‐\frac{b_{2}}{b_{1}}R_{1}(0)\right\}e^{‐c_{1}t}.$$

Therefore, we obtain,

$$I_{2}\left(t\right)‐\frac{b_{2}}{b_{1}}R_{1}\left(t\right)\to 0\;\text{for}\;t\gg \frac{1}{c_{1}}=t_{2},$$

and, since we set *I*_2_(0) = *R*_1_(0) = 0 for the initial values, we have,

(12) $$I_{2}\left(t\right)=\frac{b_{2}}{b_{1}}R_{1}\left(t\right).$$

With Equation (12), the SIIR model becomes four‐variable system. Now, we rewrite the basic equations and have,

(13) $$\frac{dS(t)}{\mathrm{dt}}=‐\beta S\left(t\right)\left(I_{1}\left(t\right)+I_{2}\left(t\right)\right)+d_{1}R_{2}\left(t\right),$$

(14) $$\frac{dI_{1}\left(t\right)}{\mathrm{dt}}=\beta S\left(t\right)\left(I_{1}\left(t\right)+I_{2}\left(t\right)\right)‐\left(b_{1}+b_{2}\right)I_{1}\left(t\right),$$

(15) $$\frac{dI_{2}\left(t\right)}{\mathrm{dt}}=b_{2}I_{1}\left(t\right)‐c_{1}I_{2}\left(t\right),$$

(16) $$\frac{dR_{2}\left(t\right)}{\mathrm{dt}}=\left(c_{1}+c_{2}\frac{b_{1}}{b_{2}}\right)I_{2}\left(t\right)‐d_{1}R_{2}\left(t\right).$$

Then, the conservation quantity becomes,

(17) $$1=S\left(t\right)+I_{1}\left(t\right)+\left(1+\frac{b_{1}}{b_{2}}\right)I_{2}\left(t\right)+R_{2}\left(t\right)+R_{3}\left(t\right).$$

For simplicity, we set *d*_1_ = 0 for now. And with Equation (12), Equations (6) and (16) are rewritten as,

$$\frac{dR_{3}\left(t\right)}{\mathrm{dt}}=c_{3}R_{1}\left(t\right)=\frac{b_{1}c_{3}}{b_{2}}I_{2}\left(t\right),\;\frac{dR_{2}\left(t\right)}{\mathrm{dt}}=\frac{b_{1}c_{2}+b_{2}c_{1}}{b_{2}}I_{2}\left(t\right).$$

By subtracting one from the other, we have,

$$\frac{d}{\mathrm{dt}}\left\{R_{3}(t)‐\frac{b_{1}c_{3}}{b_{1}c_{2}+b_{2}c_{1}}R_{2}(t)\right\}=0\;\to \;R_{3}(t)‐\frac{b_{1}c_{3}}{b_{1}c_{2}+b_{2}c_{1}}R_{2}(t)=R_{3}(0)‐\frac{b_{1}c_{3}}{b_{1}c_{2}+b_{2}c_{1}}R_{2}(0)=0.$$

where the initial values, *R*_2_(0) = *R*_3_(0) = 0, are used. Thus,

$$R_{2}\left(t\right)+R_{3}\left(t\right)=\frac{c_{1}}{b_{1}t_{1}c_{3}}R_{3}\left(t\right),$$

is obtained and the conserved quantity becomes,

(18) $$1=S\left(t\right)+I_{1}\left(t\right)+\left(1+\frac{b_{1}}{b_{2}}\right)I_{2}\left(t\right)+\frac{c_{1}}{b_{1}t_{1}c_{3}}R_{3}\left(t\right).$$

Here, if *t* → ∞, from Equations (13), (14), (15), and (16), we have,

$$‐\beta S_{*}\left(I_{1*}+I_{2*}\right)=0,$$

$$\beta S_{*}\left(I_{1*}+I_{2*}\right)‐\left(b_{1}+b_{2}\right)I_{1*}=0,$$

$$b_{2}I_{1*}‐c_{1}I_{2*}=0,$$

$$\left(c_{1}+c_{2}\frac{b_{1}}{b_{2}}\right)I_{2*}=0.$$

where *I_i_* (∞) = *I_i_*_∗_ and so on. The solutions are given by,

(19) $$I_{1*}=I_{2*}=0,\;S_{*}=\frac{\left(b_{1}+b_{2}\right)c_{1}}{\beta \left(c_{1}+b_{2}\right)}=\frac{c_{1}}{\beta t_{1}\left(c_{1}+b_{2}\right)},$$

and

(20) $$R_{3*}=\frac{c_{3}t_{1}}{c_{1}}b_{1}\left(1‐\frac{c_{1}}{\beta t_{1}(c_{1}+b_{2})}\right)\;=\frac{c_{3}t_{1}}{c_{1}}b_{1}\left(1‐\frac{c_{1}}{R_{0}(c_{1}+b_{2})}\right)$$

where,

$$R_{0}=\beta S_{0}t_{1}≃\beta t_{1}$$

is used.

In the case where herd immunity is realized in the SIIR model, what are left finite at the end of the infection process are susceptible population, recovered population with antibody, and fatalities. Because cumulative recovered population density can be expressed through cumulative fatalities density, we have,

Cumulative fatalities∝(1‐susceptible population)=Cumulative density of the infected population.

The cumulative fatalities will have a maximum value as a function of *b*_1_. The coefficient *b*_1_ is the contribution from *I*_2_(*t*) and *R*_1_(*t*) through *R*_2_(*t*), and the contribution from *R*_1_(*t*) through *R*_3_(*t*). The coefficient *b*_1_ in the cumulative density of the infected population is a contribution from *I*_2_(*t*).

The *b*_1∗_ maximizing *R*_3∗_ is found as follows. Differentiating *R*_3∗_ with respect to *b*_1_ and putting the resultant equation to 0, we have,

$$\frac{dR_{3*}}{db_{1}}=\frac{c_{3}t_{1}}{c_{1}}\left\{1‐\frac{c_{1}}{R_{0}}\left(\frac{1}{c_{1}+b_{2}}+\frac{b_{1}}{{\left(c_{1}+b_{2}\right)}^{2}}\right)\right\}=0,$$

which is solved to give,

$$b_{1*}=\frac{1}{t_{1}}‐b_{2*}=\frac{1}{t_{1}}+\frac{1}{t_{2}}\left\{1\pm \sqrt{\frac{1}{R_{0}}\left(1+\frac{t_{2}}{t_{1}}\right)}\right\}.$$

From the condition *b*_1_ < 1/*t*_1_, *b*_1∗_ is obtained as follows:

(21) $$b_{1*}=\frac{1}{t_{1}}‐\frac{1}{t_{2}}\left(\sqrt{\frac{t_{1}+t_{2}}{R_{0}t_{1}}}‐1\right).$$

It is confirmed by Equation (20) that the cumulative fatality density *R*_3∗_ in the final state is an increasing function of the fatality rate *c*_3_. Also, if the onset period (1/*c*_1_) becomes shorter, the recovery rate increases, and *R*_3∗_ is a decreasing function of *c*_1_.

### 2.6 Comparison between SIIR model and SIR model

A major feature of the SIIR model is that it considers the existence of asymptomatic individuals, namely, *I*_2_(*t*), and certainly not for the conventional models such as SIR^1^ that is defined as follow:

(22) $$\frac{dS(t)}{\mathrm{dt}}=‐\beta S\left(t\right)I\left(t\right),$$

(23) $$\frac{dI(t)}{\mathrm{dt}}=\beta S\left(t\right)I\left(t\right)‐\gamma I\left(t\right)=\left(\beta S\left(t\right)‐\gamma \right)I\left(t\right),$$

(24) $$\frac{dR(t)}{\mathrm{dt}}=\gamma I\left(t\right).$$

SIR model analyzes three transitioning stages (susceptibility (susceptible population *S*(*t*)), infectious (infectious population *I*(*t*)), and recovered (and removed) (recovered population *R*(*t*))) where infection and recovery rates are denoted as *β* and *γ*, respectively. Comparison between SIIR model and SIR model is shown in Table 1.

**Table 1:** Comparison table for SIIR model and SIR model

Items	SIIR model	SIR model
Variables	6	3
Incubation period	*t*_1_	None
Onset period	*t*_2_	1/*γ*
Asymptomatic individuals	Included	Not included
Basic reproduction number $R_{0}$	*βS*_0_ *t*_1_	*βS*_0_/*γ*
Effective reproduction number $R_{e}\left(t\right)$	$R_{0}$ (*S*(*t*)/*S*_0_)(1 + *I*_2_(*t*)/*I*_1_(*t*))	$R_{0}$ *S*(*t*)/*S*_0_
Herd immunity threshold (HIT)	1 − (1/$R_{0}$)(1 + *I*_1_(*t*)/*I*_2_(*t*))	1 − 1/$R_{0}$
Antibody acquisition	All recovered individuals	All recovered individuals
Antibody duration	1/*d*_1_	∞
Infection peak	Single and multiple peaks	Single peak

If a route which connects presymptomatic individuals with asymptomatic individuals is closed and *b*_2_ = 0 is set, *I*_2_(*t*) = *I*_2_(0)*e* ^−^ *^c^* ^1^ *^t^* in the SIIR model disappears. At the same time, if the antibody duration of the recovered individuals is set as infinity (*d*_1_ = 0) and *R*_1_ + *R*_2_ +*R*_3_ = *R* is set, SIIR model is reduced to be SIR model. Thus, it can be said that SIR model treats those who are removed from the infection networks equally and ignores internal structures. Given a finite antibody duration, these who recovered and gained antibody will be included in the group of susceptible individuals over time, but then, this will be excluded from the SIR treatment.

If we take the ratio of the basic reproduction numbers of SIR and SIIR models, we have,

$$\frac{R_{0}\left(\text{SIIR}\right)}{R_{0}\left(\text{SIR}\right)}=\gamma t_{1}.$$

Since two models may give different basic reproduction numbers for the same infectious disease, policymakers should pay attention to the model they adopt.

## 3 VERIFICATION ON SIIR MODEL BY DATA

Data used here are collected from the daily number of confirmed cases of COVID‐19 determined by PCR or antigen tests and announced daily by the prefectural health centers.^11^ The variable to be verified by the data is the daily symptomatic and quarantined population in the SIIR model which is denoted by ∆*R*_1_(*t*). This is because, among the infected population (presymptomatic, symptomatic, and asymptomatic population), presymptomatic and asymptomatic population have been rarely tested in Japan. Since the observed daily data are under uncontrolled daily fluctuations, ∆*R*_1_(*t*) = *b*_1_ *I*_1_(*t*) calculated from the SIIR model is deviated a bit from the observed daily data. Therefore, we also compare the cumulative confirmed cases of COVID‐19 with the cumulative symptomatic and quarantined population ∑∆*R*_1_(*t*) since the cumulative data are almost free from daily fluctuations which are smoothed out by coarse graining.

Below, the results of the SIIR model are compared with the observed data from Japan. Since the population size of the SIIR model is normalized, the population size of the targeted system must be determined from the data. Now, denoting the number of daily confirmed cases at date *k* by *g*(*k*) and assuming *N* to be the population size of the targeted system. We look for *N* that minimizes the following equation:

(25) $$\sum_{k=1}^{K_{d}}{\left(Nb_{1}I_{1}\left(k\right)‐g\left(k\right)\right)}^{2}$$

where *K_d_* is the total number of data. Although *I*_1_(*k*) depends on *β*, *t*_1_, *t*_2_, *b*_1_, and *I*_1_(0), *t*_1_ and *t*_2_ can be fixed as epidemiological parameters.^12^, ^13^ From the basic reproduction number $R_{0}$, which is obtained from the initial growth curve of the data, *β* can be determined as $\beta =R_{0}/t_{1}$. Also, *b*_1_ can be estimated from the position of the peak of the data. In addition, the initial value *I*_1_(0) determines the date when the amplitude rises. Thus, *N* minimizing Equation (25) is calculated as follows:

(26) $$N=\frac{\sum_{k=1}^{K_{d}}I_{1}\left(k\right)g\left(k\right)}{\sum_{k=1}^{K_{d}}b_{1}I_{1}{\left(k\right)}^{2}}.$$

Here, note that, even if *b*_1_ *I*_1_(*k*) and *g*(*k*) are similar in profiles, Equation (25) is not necessarily minimized, as long as the date when the amplitude rises are different. Therefore, Equation (26) is used to calculate the population size of the system, and *I*_1_(0) minimizing Equation (25) for given *N* is numerically calculated.

There are many places of close contacts almost everywhere in Japan. Infections in a group of susceptible population associated with such places could “fire up” separately and simultaneously, forming a peak of infection. If, after the end of the outbreak prevention policy, the infection is not completely terminated, the spread of infection can start again from new close contact between remaining infected individuals and susceptible individuals. Then, following a similar process to the formation of the first peak, peaks of newly infected individuals appear repeatedly with the period of policy. If policies like self‐restraint requests, closures of schools and restraints on conducting business are made nation‐widely in a uniform manner without considering the locality of the infection spread, a larger infection spread caused by “fade out of caution” is likely to happen after the policies are lifted.

Extending the SIIR model to describe this chain of spreads is not really difficult. We can build a scenario where remaining presymptomatic and asymptomatic carriers connect several SIIR systems. In fact, it seems that those carriers are moving to discover new areas of spread. It is possible to create *n* chained SIIR systems and consider that after the first system has ignited and terminated, the remaining presymptomatic and asymptomatic carriers will drive the spread as the initial value for the second system.

Regarding the characteristic values of infectious disease, according to the WHO site, in April 2, 2020, the incubation period of COVID‐19 is 5‐6 days on average, mainly based on data from Wuhan, and it is infectious during the incubation period.^14^ Lauer et al also reported that the median incubation period of COVID‐19 was 5.1 days (95% CI, 4.5‐5.8 days) and that the infected individuals will become symptomatic within 11.5 days with 97.5% (95%CI, 8.2‐15.6 days).^12^ It seems that no definitive data analysis has been conducted regarding the onset period, however, according to a survey report by the National Institute of Infectious Diseases (March 23, 2020) in the guidelines of the Ministry of Health, Labor and Welfare in Japan in April 22, 2020, the average length of hospitalization is 16.6 days.^13^ So, the following values,

$$t_{1}=5,\;t_{2}=17,$$

are used in the numerical calculations of the SIIR model. The mortality rate *c*_3_ is set according to the fatalities in the data, and *d*_1_, which is the inverse of the antibody duration, is set as *d*_1_ = 0 since there are no evidence data at this moment though several cases are reported from several countries.

**Figure 3:**
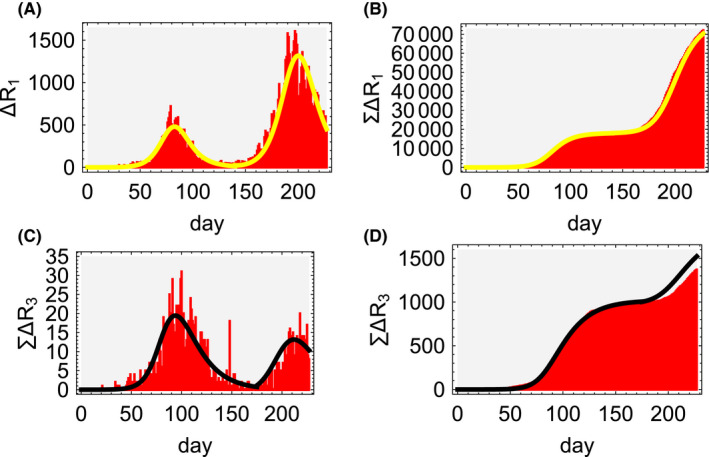
Upper left: the number of confirmed cases in Japan (data from January 24 to September 6, 2020: red) and that of new symptomatic and quarantined individuals *b*_1_ *I*_1_(*t*) (SIIR model: yellow). Upper right: the cumulative number of confirmed cases in Japan (red) and that of new symptomatic and quarantined individuals ∑*_t_* *b*_1_ *I*_1_(*t*) (SIIR model: yellow). Lower left: daily fatalities by COVID‐19 in Japan (red) and *c*_3_ *R*_1_(*t*) (SIIR model: black). Lower right: cumulative fatalities by COVID‐19 in Japan (red) and ∑*_t_* *c*_3_ *R*_1_(*t*) (SIIR model: black)

Figure 3 (upper row) shows the number of confirmed cases and the cumulative number of confirmed cases in Japan (red) obtained from data and the corresponding number of new symptomatic and quarantined individuals in the SIIR model (yellow).

Here, two normalized SIIR systems $A_{1}$ and $A_{2}$ are connected at the day *K* = 140 to describe the spread and termination of infection observed in Japan:

$$A_{i}=\left\{S^{\left(i\right)}\left(t\right),I_{1}^{\left(i\right)}\left(t\right),I_{2}^{\left(i\right)}\left(t\right),R_{1}^{\left(i\right)}\left(t\right),R_{2}^{\left(i\right)}\left(t\right),R_{3}^{\left(i\right)}\left(t\right)\right\},\;i=1,2,$$

$$1=S^{\left(i\right)}\left(t\right)+I_{1}^{\left(i\right)}\left(t\right)+I_{2}^{\left(i\right)}\left(t\right)+R_{1}^{\left(i\right)}\left(t\right)+R_{2}^{\left(i\right)}\left(t\right)+R_{3}^{\left(i\right)}\left(t\right).$$

The parameters of $A_{1}$ are set as follows:

(27) $$t_{1}=5,\;t_{2}=17,\;\beta =0.16,$$

(28) $$b_{1}=\frac{0.06}{t_{1}},\;b_{2}=\frac{1}{t_{1}}‐b_{1},\;c_{1}=\frac{1}{t_{2}},\;c_{2}=\frac{0.942}{t_{2}},c_{3}=\frac{1}{t_{2}}‐c_{2},\;d_{1}=0.$$

The initial values of $A_{1}$ are as follows:

(29) $$S^{\left(1\right)}\left(0\right)=1‐I_{1}^{\left(1\right)}\left(0\right),\;I_{2}^{\left(1\right)}\left(0\right)=R_{1}^{\left(1\right)}\left(0\right)=R_{2}^{\left(1\right)}\left(0\right)=R_{3}^{\left(1\right)}\left(0\right)=0.$$

Then, the following values,

$$N_{1}=316067,\;I_{1}\left(0\right)=0.00004,$$

are obtained from Equations (25) and (26). The total number of observation data is set as *K* = 140 days.

After the spread of infection in system $A_{1}$ met the condition of herd immunity and settled down, some remaining infectious individuals in $A_{1}$ contacted susceptible individuals in system A_2_ and caused spread of infection in $A_{2}$. This relay of infections from $A_{1}$ to $A_{2}$ happened at *t* = *K* (=140). The parameters for $A_{2}$ are taken to be the same as Equations (27) and (28) in $A_{1}$. The initial values at *t* = *K* are set as follows:

(30) $$S^{\left(2\right)}\left(K\right)=1‐I_{1}^{\left(2\right)}\left(K\right),\;I_{2}^{\left(2\right)}\left(K\right)=R_{1}^{\left(2\right)}\left(K\right)=R_{2}^{\left(2\right)}\left(K\right)=R_{3}^{\left(2\right)}\left(K\right)=0,$$

where the population size of $A_{2}$ is calculated from Equations (25) and (26) as follows:

$$N_{2}=1060790,\;I_{1}^{\left(2\right)}\left(K\right)=0.002.$$

Note that the basic reproduction number here is $R_{0}$ = 0.79968 (<1), and the infection does not spread in the SIR model; however, it spreads in the SIIR model as shown in Equation (8).

The asymptomatic population can be obtained from the SIIR models and data. From Equation (12), it can be seen that the asymptomatic population *I*_2_(*t*) is proportional to the symptomatic and quarantined population *R*_1_(*t*) and with the given parameters the ratio is given by,

$$\frac{I_{2}\left(t\right)}{R_{1}\left(t\right)}=\frac{b_{2}}{b_{1}}=15.67.$$

In other words, the asymptomatic population in Japan can be approximately 16 times greater than symptomatic and quarantined population. Data also show that asymptomatic individuals who had refrained from going out during the state of emergency declaration went out again after it was lifted, made new contacts, and spread of infection. From this, a major factor leading to the spread of infection could be understood because of the fact that the main target of the PCR test in Japan has been limited to people with subjective symptoms on COVID‐19. It is said that the reason for limiting the PCR test in Japan was to prevent medical collapse. However, it is necessary to verify whether the policies taken with the supplementary budget should have been directed to prevent medical collapse.

Now, we take a close look at fatalities caused by COVID‐19 in Japan, which can be reproduced quite well with the SIIR model (Figure 3 (lower row). Since *R*_1_ and *R*_3_ are on different stages, there is a difference between the timings when the peaks occur. Therefore, in Figure 3 (lower row), the time when $A_{1}$ connects to $A_{2}$ is set as *K* = 170, which is different from *K* = 140 used in Figure 3 (upper row), and the fatality rates are set as follows:

$$c_{3}=\left\{\begin{array}{l} \frac{0.058}{t_{2}}\;\text{for}\;t\leq K,\\ \frac{0.014}{t_{2}}\;\text{for}\;t>K. \end{array}\right)$$

In $A_{2}$, unlike $A_{1}$, more of the infected are young, fewer elderly people with preexisting medical conditions are infected, and, in addition, healthcare professionals have gained experience in $A_{1}$ so that they can respond appropriately to treatment. However, in the future, if the infection spreads from the young to the elderly, and if the “GO TO TRAVEL” campaign in Japan boosts the spread of infection to more people including those with preexisting medical conditions and the elderly, then fatalities may also increase. Finding and isolating asymptomatic individuals can help control the spread of the infection.

## 4 DISCUSSIONS

In this paper, the SIIR model, dealing with the features of COVID‐19 such as presymptomatic individuals who have infectivity even during the incubation period, asymptomatic individuals who can freely move around and play crucial roles in the spread of infection, and limited duration of immunity, has been introduced to show that it can replicate data such as the number of confirmed cases for COVID‐19 and that of those who passed away because of COVID‐19 in Japan.

Since infectious diseases spread depending on individual's health conditions and behavioral characteristics, the SIIR model cannot provide detailed information for policymaking, as far as our discussion is limited within median or mean. This is the limit of the SIIR model. In the future, we will address the following topics in order to discuss policy issues for the early termination of COVID‐19.

- Verification of the SIIR model based on the spread of COVID‐19 infection in various countries.
- Formulation of agent‐based modeling (ABM) that incorporates diversity such as individual health conditions and behavioral characteristics and verification of ABM by actual data.
- Examination of detailed policies corresponds toward the convergence of infectious diseases.

## CONFLICT OF INTEREST

The authors have stated explicitly that there are no conflicts of interest in connection with this article.

## AUTHOR CONTRIBUTIONS

All authors had access to the data and a role in writing the manuscript.

## INFORMED CONSENT

There is no need to obtain any consent for this article.
